# Development of an alginate–chitosan biopolymer composite with dECM bioink additive for organ-on-a-chip articular cartilage

**DOI:** 10.1038/s41598-024-62656-1

**Published:** 2024-05-23

**Authors:** Upasna Upadhyay, Saketh Kolla, Siddhartha Maredupaka, Swapna Priya, Kamma Srinivasulu, Lakshmi Kiran Chelluri

**Affiliations:** 1 Stem Cell Unit, Global Medical Education and Research Foundation (GMERF), Lakdi-ka-pul, Hyderabad, Telangana 500004 India; 2 Department of Biotechnology, Koneru Lakshmaiah Education Foundation (KLEF) Deemed to be University, Vaddeswaram, Vijayawada, Andhra Pradesh 522302 India; 3 Department of Orthopaedics, Gleneagles Global Hospitals, Lakdi-ka-pul, Hyderabad, Telangana 500004 India; 4 Advanced Diagnostics and Therapeutics, Gleneagles Global Hospitals, Lakdi-ka-pul, Hyderabad, Telangana 500004 India; 5 Academics and Research, Global Medical Education and Research Foundation (GMERF), Gleneagles Global Hospitals, Lakdi-ka-pul, Hyderabad, Telangana 500004 India

**Keywords:** Stem cells, Adult stem cells, Mesenchymal stem cells, Stem-cell differentiation, Biomimetics, Tissue engineering

## Abstract

In vitro use of articular cartilage on an organ-on-a-chip (OOAC) via microfluidics is challenging owing to the dense extracellular matrix (ECM) composed of numerous protein moieties and few chondrocytes, which has limited proliferation potential and microscale translation. Hence, this study proposes a novel approach for using a combination of biopolymers and decellularised ECM (dECM) as a bioink additive in the development of scalable OOAC using a microfluidic platform. The bioink was tested with native chondrocytes and mesenchymal stem cell-induced chondrocytes using biopolymers of alginate and chitosan composite hydrogels. Two-dimensional (2D) and three-dimensional (3D) biomimetic tissue construction approaches have been used to characterise the morphology and cellular marker expression (by histology and confocal laser scanning microscopy), viability (cell viability dye using flow cytometry), and genotypic expression of ECM-specific markers (by quantitative PCR). The results demonstrated that the bioink had a significant impact on the increase in phenotypic and genotypic expression, with a statistical significance level of *p* < 0.05 according to Student’s *t*-test. The use of a cell-laden biopolymer as a bioink optimised the niche conditions for obtaining hyaline-type cartilage under culture conditions, paving the way for testing mechano-responsive properties and translating these findings to a cartilage-on-a-chip microfluidics system.

## Introduction

Cartilage tissue engineering has been explored for the development of advanced therapeutic approaches to restore normal functional and structural properties^1^. The prevailing strategies in cartilage tissue engineering are meant to closely emulate the native environment and the gradient structure of chondrocytes to promote the restoration of their extracellular matrix (ECM)^2,3^. Two-dimensional (2D) platforms derived from primary chondrocytes, mesenchymal stem cell (MSC)-induced chondrocytes^4^ or chondrogenic cell lines^5^ provide a quick and cost-effective approach for studies in cartilage tissue engineering. However, the well-established phenomenon of dedifferentiation in chondrocytes shifting from a rounded to a more fibroblastic structure as a monolayer culture is a major downside closely resembling fibrocartilage rather than hyaline cartilage^6^.

In efforts to increase the physiological significance of 2D models and establish micro-physiological 3D models, researchers have incorporated decellularised ECM (dECM) to mimic both mechanical and chemical properties^7^. Cartilage-derived native dECM has been demonstrated to facilitate chondrocyte adhesion, growth and proliferation and to enhance chondrogenic differentiation of bone marrow-derived mesenchymal stem cells (BMSCs) in vitro for cartilage regeneration^8^. Furthermore, this involves the exploration and integration of biopolymers such as collagen^9^, gelatin^10^, silk fibroin^11^, fibrin^12^, chitosan^13^, alginate^14^, and hyaluronan^15^. An ideal biopolymer construct should possess biocompatibility, biodegradability, chemical stability, and ease of polymerisation, making it suitable for demonstrating phenotypic, genotypic, and mechanoresponsive properties^16^. Alginate natural biopolymers serve as three-dimensional structures to mimic the ECM, offering a supportive environment for cell growth and proliferation owing to their adjustable porosity and facile polymerisation with divalent cations. Cellular responses to alginate biopolymers include the expression of collagen type II, aggrecan and glycosaminoglycan (GAG) deposition^17^. However, limitations exist, such as the lack of cell adhesion sites, limited mechanical strength, potential for rapid degradation, and inconsistent gelation owing to varying concentrations. These challenges have been addressed through appropriate modifications and combinations with other hydrogels^18^.

Chitosan, a versatile natural biopolymer, has emerged as the preferred choice for fabricating hydrogels to replicate multi-layered gradient zones of cartilage with specific ECM chemical compositions and mechanical properties. It has a chemical structure similar to that of GAG in native cartilage tissue. The inherent properties of biocompatibility, biodegradability, muco-adhesivity, and antimicrobial activity^19^ make chitosan a promising candidate for rapid mechanical recovery under compressive loading, and it remains intact under tensile loading without delamination^20^. Although chitosan is tough and flexible, it lacks the strength required for use alone in load-bearing applications. This limitation has been addressed using glutaraldehyde, which can crosslink chitosan-free amine groups^21^. Furthermore, combining alginate with chitosan provides strength, making it ideal for applications that require robust structural integrity.

The combination of alginate and chitosan reportedly improves cell attachment and proliferation in 3D culture environments, favouring a typical rounded cell morphology, as observed in the chondrogenic lineage^22^. Additionally, the amino groups of chitosan can provide cell adhesion sites, addressing one of the limitations of alginate. The specific properties and performance of alginate–chitosan composite hydrogels can be influenced by factors such as the ratio of alginate to chitosan, the degree of crosslinking, and any additional modifications. The alginate–chitosan composite also contributes to the mechanical stability of the hydrogel, making it suitable for mechanical stimulation, cell-laden hydrogels, microchannels for nutrient delivery, and chambers for dynamic culture^23^. This integration allows a more comprehensive representation of the cartilage microenvironment using a microfluidic system to overcome challenges in terms of fabrication, ensuring dynamic culture conditions, and designing channels with microscale dimensions using appropriate material properties.

The current work elucidates the optimisation of alginate and chitosan composite hydrogel. It further emphasises the role of the bioink additive on the articular cartilage tissue construct.

## Results

### Characterisation of bioink using confocal laser scanning microscopy (CLSM)

**Figure 1:**
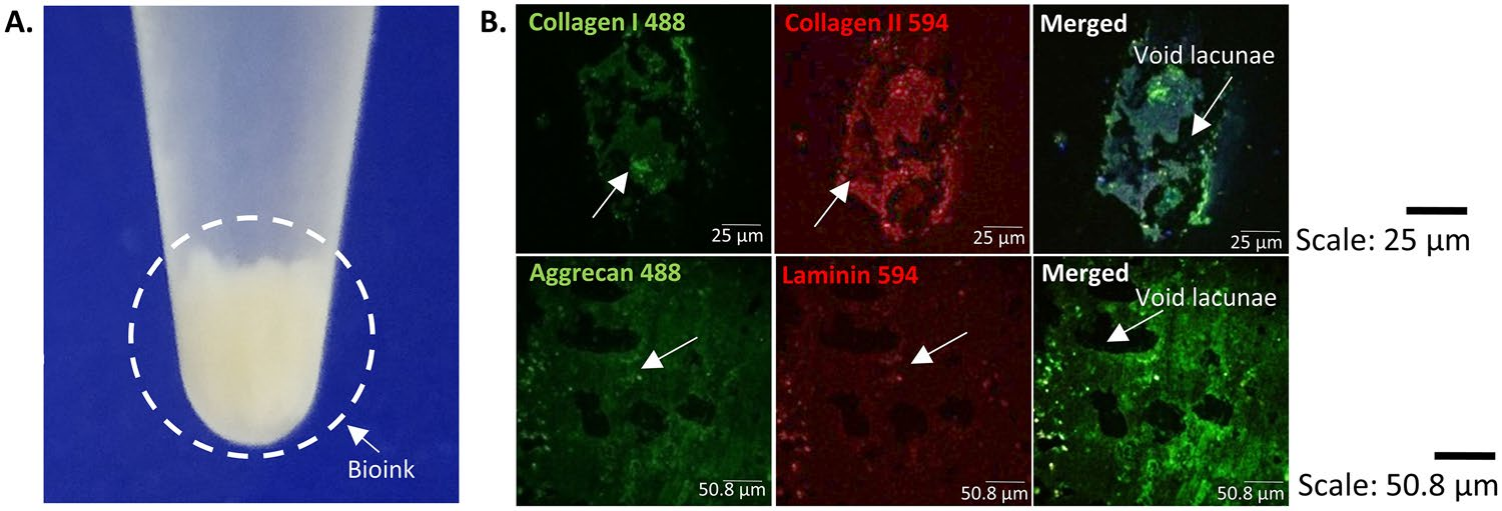
Bioink and its characterisation. (**A**) White buffy cell-free bioink obtained from native articular cartilage. (**B**) Cell-free bioink characterised for conservation of ECM markers showing positive expression of collagen type I, collagen type II, aggrecan and negligible expression of laminin as a suitable niche for integration with biomimetic tissue constructs.

The native articular cartilage post 16 h decellularisation resulted in white, opaque, buffy bioink additive (Fig. 1A). The bioink additive characterised for efficient decellularisation resulted in cell-free ECM with void lacunae. The cell-free ECM characterisation also revealed positive expression for collagen type I, collagen type II, and aggrecan ECM specific markers with minimal expression of cell-ECM laminin marker (Fig. 1B).

### 2D plate culture characterisation

**Figure 2:**
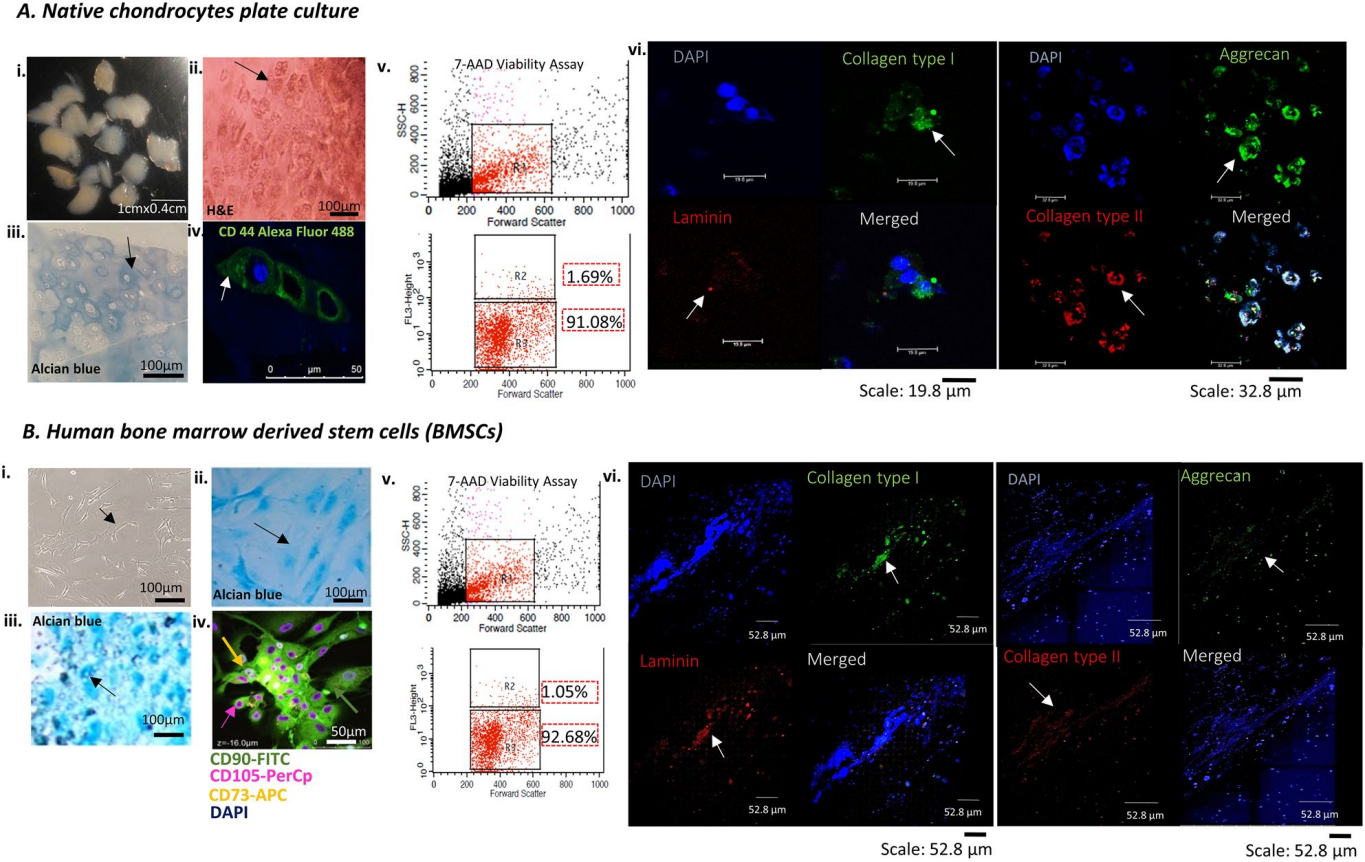
2D plate characterisation of human tissue sources. The figure summarises (**A**) native chondrocytes (i) isolation and culturing from articular cartilage explants highlighting (ii) three-weeks culture H&E staining for morphological identification (iii) alcian blue staining for GAG accumulation around cells (iv) and chondrocyte specific CD44 marker positive expression for phenotype characterisation (v) flow cytometry viability assay showed R1 representing as chondrocytes population, R2 as dead cell population and R3 as live cell population (vi) CLSM characterisation showed positive expression for ECM-specific markers collagen type I, type II, aggrecan with negligible expression for laminin cell-ECM marker. (**B**) BMSCs-induced chondrocytes characterisation showed (i) bright field images of cultured BMSCs with spindle shaped morphology (ii) alcian blue staining showed GAG accumulation for undifferentiated state of BMSCs (iii) and in differentiated state of chondrogenesis (iv) CLSM characterisation showed positive expression for BMSCs specific surface markers CD90-FITC, CD73-APC, CD105-PerCp (v) flow cytometry characterisation showed percentage viability for live cell population represented as R3 and (vi) CLSM characterisation for differentiated chondrocytes showed positive expression for ECM-specific marker collagen type I, aggrecan and laminin with minimal expression for collagen type II. H&E- Haematoxylin and eosin; GAG- glycosaminoglycan; ECM-extracellular matrix; CLSM-confocal laser scanning microscopy; 7AAD-7 aminoactinomycin; ICC-immunocytochemistry; DAPI- 4',6-diamidino-2-phenylindole; BMSCs-bone marrow-derived mesenchymal stem cells; FITC-fluorescein isothiocyanate; APC-allophycocyanin; PerCp-peridinin chlorophyll protein.

The isolated cells from native cartilage explants cultured and stained with haematoxylin and eosin (H&E) revealed a characteristic elliptical to oval-shaped morphology of chondrocytes, which formed isogenous groups of up to four cells enclosed in lacunae embedded within the ECM (Fig. 2Ai–ii). Alcian blue staining (pH-2.5) showed ECM for its rich GAG content accumulation, indicating an essential component of articular cartilage (Fig. 2A iii). In addition, chondrocytes exhibited positive expression of CD44, characterising the cell-specific surface marker (Fig. 2A iv). The 7-aminoactinomycin D (7-AAD) viability assay revealed the total chondrocytes population R1, dead cell population of 1.69% as R2, and live cell population of 91.08% as R3 after three weeks of culture (Fig. 2A v). The chondrocytes also showed positive qualitative expression of collagen type I, collagen type II, and aggrecan, characterising the growth of fibrous cartilage under the provided culture conditions with negligible expression of laminin (Fig. 2A vi).

The BMSCs exhibited spindle-shaped morphology when cultured for 70% confluence, indicating their attachment and proliferation potential (Fig. 2B i). Alcian blue staining demonstrated the differentiation potential to chondrogenic lineage with accumulated sulphated GAG (Fig. 2Bii–iii). This is a crucial indicator of chondrogenic differentiation, suggesting the cells can form cartilage-mimicking structures. CLSM characterisation revealed positive expression for surface markers CD 90-FITC, CD 105-PerCp, and CD 73-APC (Fig. 2B iv). The 7-AAD viability assay revealed the total BMSCs-induced chondrocytes population as R1, dead cell population of 1.05% as R2, and live cell population of 92.68% as R3 after three weeks of culture (Fig. 2B v). These results confirm the phenotypic characterisation, differentiation potential of BMSCs to chondrogenic lineage and their viability. The BMSCs-induced chondrocytes also showed positive qualitative expression of collagen type I, aggrecan and laminin with minimal expression of collagen type II characterising the growth of fibrous cartilage (Fig. 2B vi). The results successfully characterised chondrocytes and BMSCs-induced chondrocytes, ensuring proper adhesion, proliferation, differentiation and laying the foundation for the development of biomimetic tissue constructs.

### 3D pellet and alginate culture characterisation without/with bioink using histology, scanning electron microscopy (SEM) and CLSM

**Figure 3:**
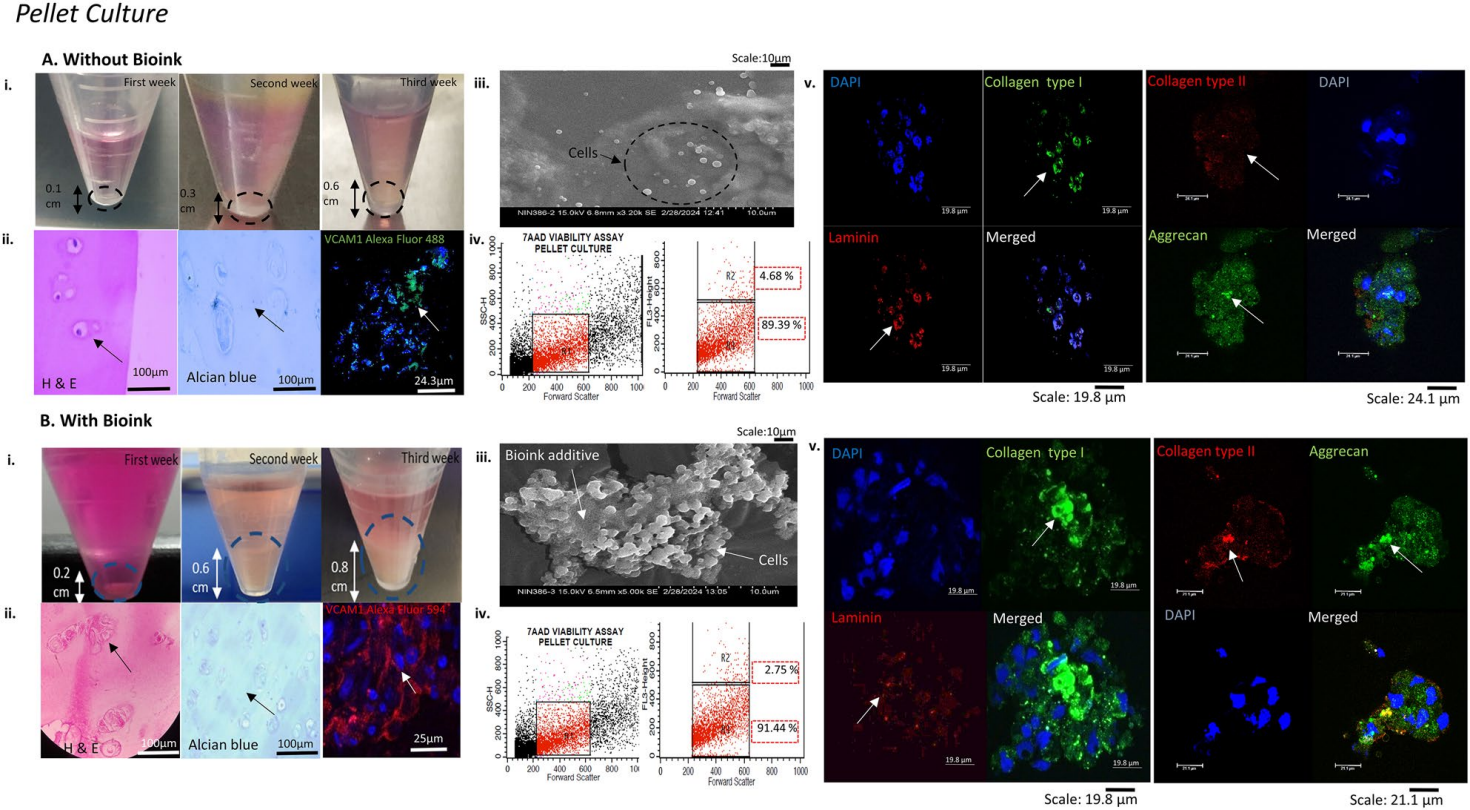
Biomimetic pellet tissue construct characterisation for BMSCs differentiated chondrocytes. The figure shows the BMSCs-induced chondrocytes characterisation (**A**) without bioink (i) cultured and monitored for increasing pellet size for three weeks (ii) H&E staining showed oval-shaped morphology with alcian blue demonstrated GAG accumulation around the cells and CLSM imaging showed BMSCs differentiation marker for chondrogenesis VCAM1 positive expression. (iii) SEM imaging showed fewer cells growing individually without bioink (iv) Cell viability analysis with 7-AAD dye using flow cytometry showed R1 as the desired population, R2 as the dead cell population, and R3 as the live cell population showing the percentage viability of the cultured construct. (v)The ECM-specific markers showed positive expression for collagen type I, aggrecan (Alexa Fluor 488 conjugated) and laminin and minimal expression for collagen type II (Alexa Fluor 594 conjugated). (**B**) With bioink (i) the pellet increased in size to 0.8 cm after three weeks of culture. (ii) H&E staining revealed isogenous clusters of cells, increased GAG content and increased expression of VCAM 1 in proliferating BMSCs-induced chondrocytes. (iii) The SEM imaging showed cellular attachment on bioink additive (iv) The 7AAD viability assay showed an increase in the percentage of the live cell population R3. (v) CLSM characterisation revealed an increase in chondrogenesis secreted markers collagen type II expression, in addition to collagen type I, aggrecan with cell-ECM marker laminin expression, and counterstaining with the nuclear dye DAPI (blue).

The pellet culture grown with bioink laden BMSCs-induced chondrocytes showed an increased pellet size of 0.8 cm in comparison to pellet culture grown without bioink (Fig. 3A–B i). The H&E staining revealed a characteristic round to polygonal morphology of BMSCs differentiated state, resembling native cartilage nodules phenotypically. The alcian blue and vascular cell adhesion molecule 1 (VCAM1) staining showed intense GAG accumulation in pericellular matrix of ECM and positive expression of BMSCs chondrogenic marker respectively with bioink (Fig. 3A–B ii). Thus, the histology and cell-specific surface marker characterisation demonstrated the formation of cartilage-like biomimetic structure. The SEM ultrastructure characterisation revealed the attachment and aggregates of BMSCs-induced chondrocytes supported on bioink, in comparison to without bioink showing few cellular growth (Fig. 3A–B iii). The 7-AAD viability assay showed significant percentage increase in live cell population (R3-91.44%) with bioink, compared to without bioink live cell population (R3-89.39%) (Fig. 3A–B iv). In addition, CLSM characterisation for pellet culture with bioink showed positive expression for collagen type II (hyaline-type cartilage specific marker), proteoglycan accumulation marker aggrecan and the cell-ECM adhesion marker laminin (Fig. 3A–B v).

**Figure 4:**
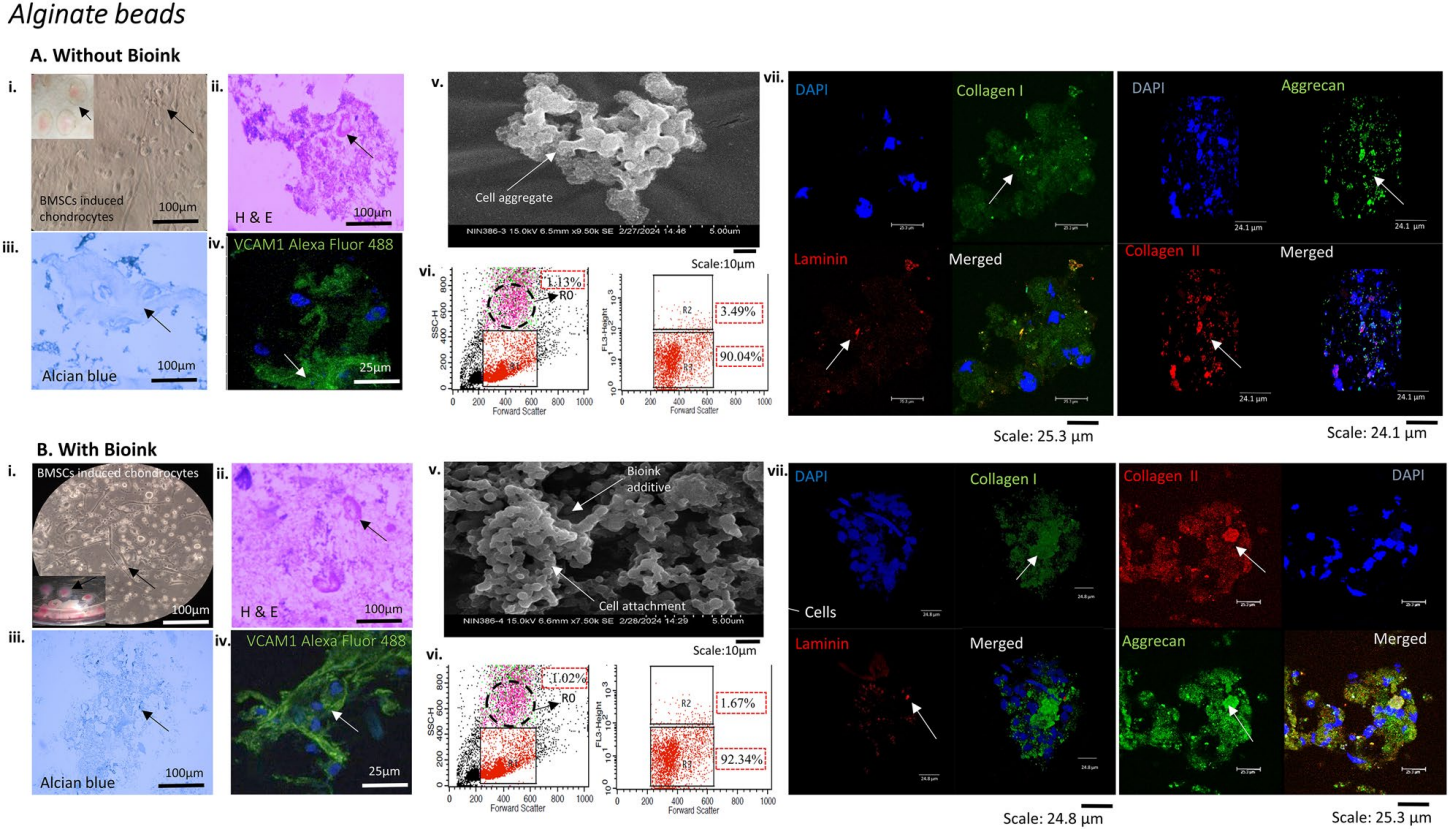
Biomimetic alginate bead tissue construct characterisation of BMSCs differentiated chondrocytes. The figure depicts the phenotype and ECM-specific marker characterisation of cultures (**A**) without bioink and (**B**) with bioink. (i) The BMSCs-induced chondrocytes were cultured as alginate beads for three weeks and monitored via light microscope showed increased confluency with bioink in comparison to without bioink. (ii) H&E characterisation confirmed the oval-shaped morphology in both without and with bioink cultures (iii) Alcian blue staining revealed the intense GAG content accumulated around differentiated chondrocytes with bioink comparative to without bioink (iv) The BMSCs-induced cells showed intense positive VCAM1 expression conjugated with Alexa Fluor 488 with bioink (v) The SEM imaging showed fewer cell aggregates within alginate bead without bioink, while the bioink additive showed comparatively more cellular attachments and aggregates (vi) Cellular viability assessed using flow cytometry with live cell population (R3) showed increased percentage viability with bioink. R0 represented hypergranular population (1.02%), not of study interest (vii) the CLSM characterisation showed positive expression with bioink for hyaline cartilage ECM-specific markers collagen type I, collagen type II, aggrecan and cell-ECM marker laminin, counterstained with the nuclear dye DAPI.

In comparison to without bioink, the alginate bead biomimetic constructs with BMSCs-induced chondrocytes laden bioink, cultured in chondrogenesis media for three weeks showed confluent attachment and growth with spherical morphology, as observed by bright field imaging and H&E staining (Fig. 4A, B i–ii). The 3D microenvironment of the alginate beads supported dense GAG accumulation for growth and matrix production with bioink in comparison to without bioink (Fig. 4A–B iii). The bioink also promoted chondrogenic differentiation of BMSCs, leading to the production of cartilage-specific matrix components, as observed by positive expression of VCAM1 characterisation (Fig. 4A–B iv). The SEM surface structure characterisation showed the attachment and aggregates of BMSCs-induced chondrocytes on bioink additive, in comparison to without bioink showing few cellular aggregates (Fig. 4A–B v). The 7-AAD viability assay showed an increased percentage of viable cell population (R3-92.34%) with bioink, indicating its potential as a niche for promoting enhanced chondrogenic differentiation compared to that of cells without bioink (R3-90.04%). Also the dead cell population (R2) percentage was reduced to 1.67% with bioink in comparison to without bioink showing higher percentage of 3.49%. The flow cytometry forward scatter showed unrelated small percentage of hypergranular population of undifferentiated cells (R0) (Fig. 4A–B vi). The CLSM characterisation with ECM-specific markers resulted in expression of collagen type I without bioink resulted in the formation of homogenous fibrous cartilage (Fig. 4A vii). While integrating bioink additive, provided a continuous chondrogenic environment shifting the ECM niche towards a more functional hyaline cartilaginous matrix, with an increase in the type II collagen expression (Fig. 4B vii).

### Cell proliferation assay

**Figure 5:**
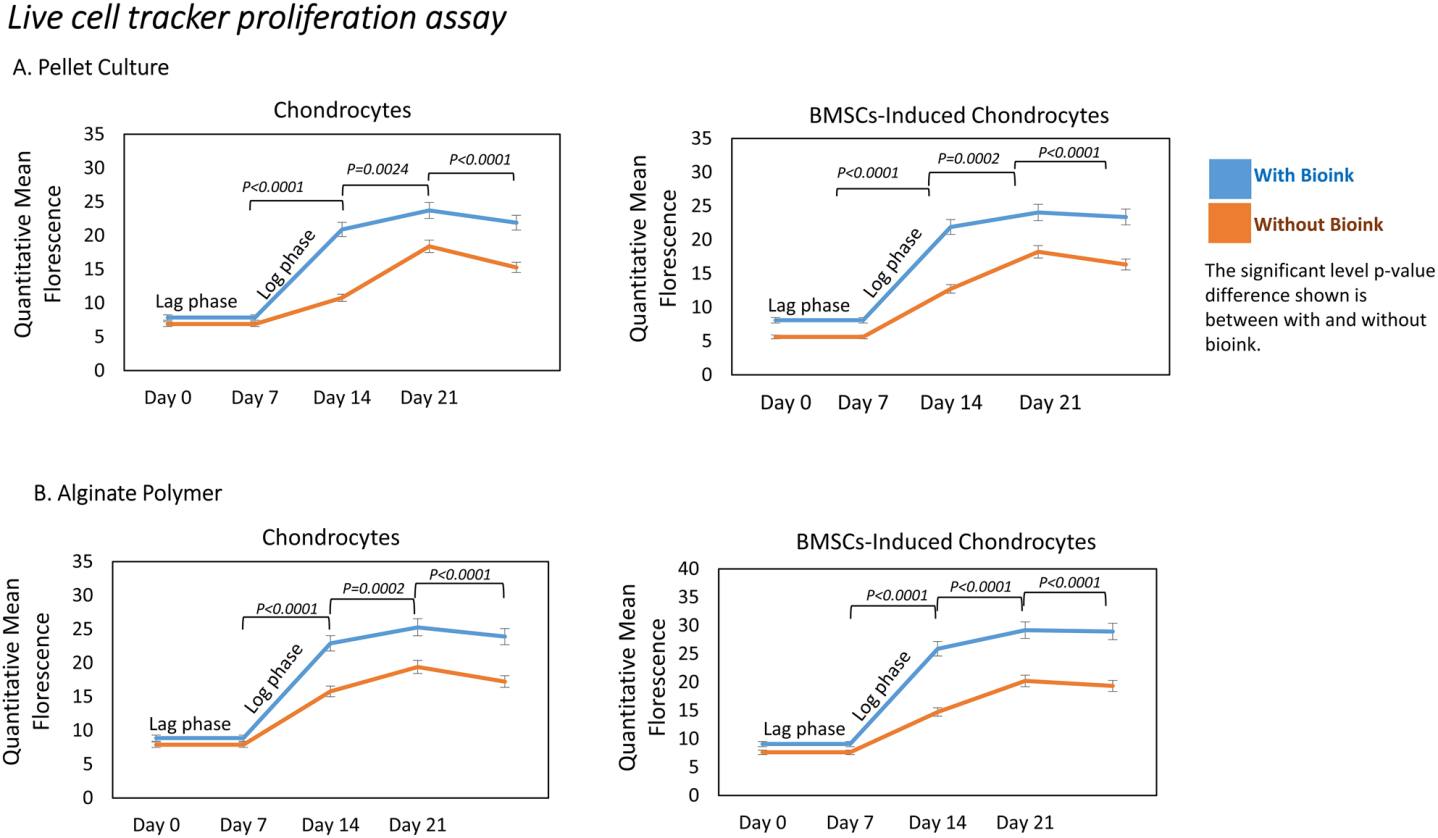
Live cell tracker proliferation assay. (**A**) The quantitative mean fluorescence for the pellet culture and (**B**) alginate bead biopolymer construct showed a sigmoid growth pattern with an initial lag phase up to seven days, followed by log phase of growth with increased proliferation rate up to 21 days with bioink for both chondrocytes and BMSCs-induced chondrocytes in comparison to without bioink 3D constructs.

The live cell tracker dye produced a sigmoid growth pattern based on the quantitative mean fluorescence. The sigmoid growth curve showed an initial lag phase followed by cells rapidly proliferating to log phase. The cells reach a plateau stage maintaining in their differentiation state up to 21 days. The mean and standard deviation (n = 10) calculated for proliferation rates over three weeks without and with bioink showed statistically significant difference with *p* < 0.05. This provided insights into the temporal dynamics of enhanced cell proliferation and their distribution in the pellet and alginate tissue constructs with bioink (Fig. 5).

### Gene expression analysis

**Figure 6:**
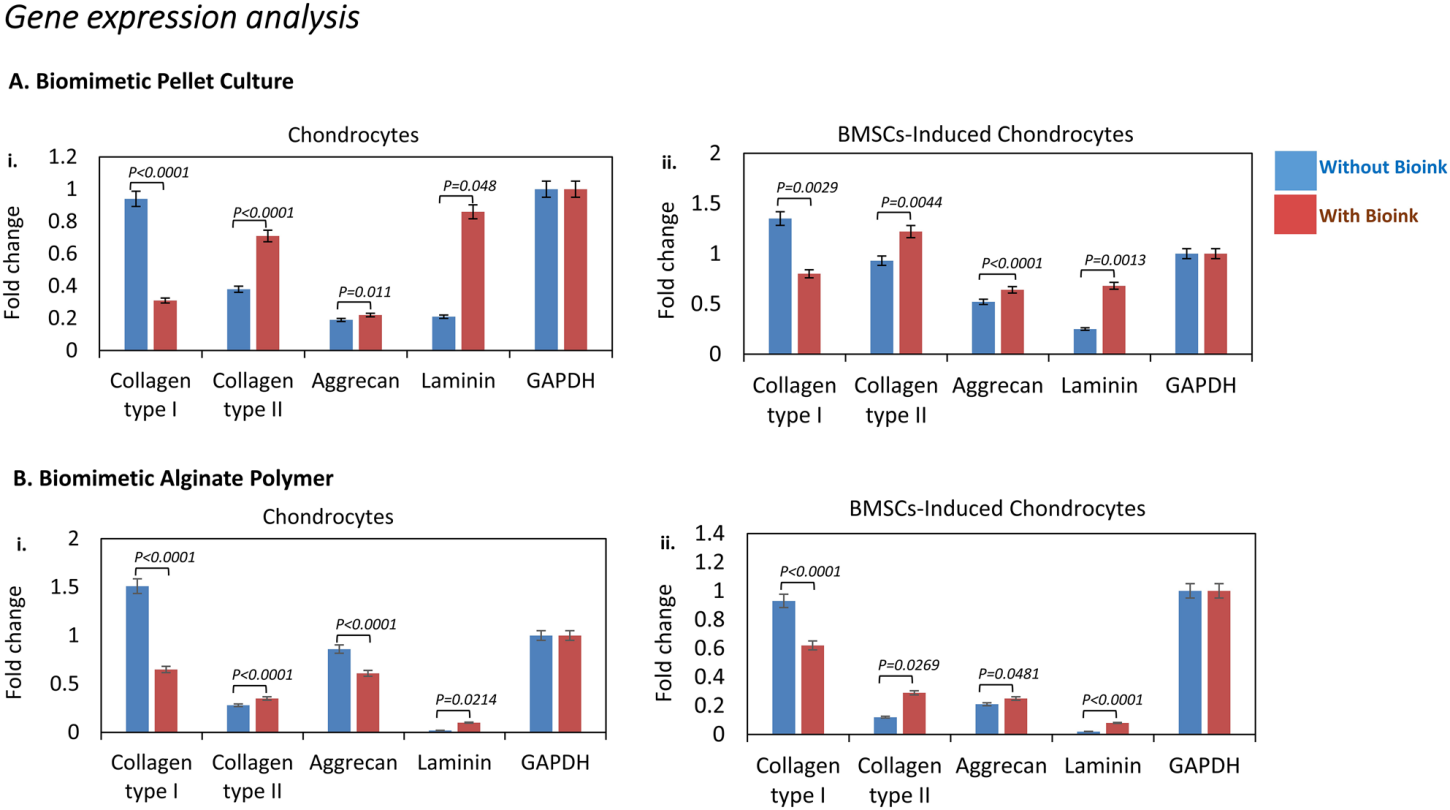
Quantitative PCR gene expression analysis (**A**, **B**) the graphs represent the fold change in the gene expression of the ECM-specific markers collagen type I, collagen type II, aggrecan and laminin normalised to the housekeeping reference gene GAPDH. (**Ai-ii**) Compared to culture without bioink, the pellet culture with bioink for chondrocytes and BMSCs-induced chondrocytes showed an increase in the gene expression of the collagen type II and laminin and a decrease in the gene expression of the collagen type I. (**Bi-ii**) The alginate biopolymer with bioink showed reduced expression of collagen type I and increased expression of collagen type II, for both chondrocytes and BMSCs-induced chondrocytes in comparison to cells without bioink. The statistical student’s t-test with calculated p-value less than significant level (α) = 0.05 was considered.

The fold change in the gene expression profile demonstrated the impact of bioink on chondrocytes and BMSCs-induced chondrocytes in pellet culture and alginate bead biopolymer. In pellet cultures without bioink, chondrocytes exhibited lower gene expression fold change of collagen type II (0.38) and aggrecan (0.19). However, the presence of bioink enhanced the gene expression fold change of collagen type II (0.71) and aggrecan (0.22) in chondrocytes, suggesting that bioink have a positive influence on the production of ECM components by chondrocytes (Fig. 6A i). BMSCs-induced chondrocytes in pellet culture without bioink showed an increase in fold change of collagen type I (1.35) and collagen type II (0.93) expression compared to that of chondrocytes, indicating a shift toward a chondrogenic lineage. With bioink, BMSCs-induced chondrocytes further enhanced collagen type II (1.22) fold change expression, reinforcing the favourable impact of bioink on BMSCs for chondrogenesis (Fig. 6A ii).

The alginate bead construct without bioink, BMSCs-induced chondrocytes exhibited various changes in gene expression, with increased fold change expression of collagen type I (0.93) and decreased gene expression of collagen type II (0.12) and aggrecan (0.21). With bioink, there was an overall improvement in BMSCs-induced chondrocytes gene expression, particularly in collagen type II (0.29) and aggrecan (0.25), indicating the positive influence of bioink on promoting a hyaline type cartilage. Furthermore, the expression of laminin, a non-chondrogenic marker, was generally low under culture conditions without bioink. The bioink additive increased laminin gene expression, confirming cellular attachment to the dECM bioink (Fig. 6B). The expression of the housekeeping gene GAPDH remained relatively constant across all conditions, providing a reliable reference for normalization.

### Alginate‒chitosan polymer composites

The increased alginate polymer concentration from 1 to 2% and its interaction with chitosan and increased concentration of cross-linking agents resulted in a denser hydrogel with increased stability, homogenous formulation, faster gelation time, and balanced physiological pH, indicating biocompatibility with cells (Table 2). Thus, the mechanoresponsive properties of the prepared hydrogel composites will be tested, and composite hydrogels resembling native articular cartilage will be considered for microscale fabrication of cartilage tissue engineering using microfluidics in our ongoing work.

## Discussion

Native articular cartilage protein profiling revealed various heterotypic collagens, proteoglycan chains of sulfated GAG, and the highly anionic hydrophilic molecule aggrecan as major composition prerequisites for cartilage ECM restoration. These findings underscore the rationale behind selecting a native bioink over a synthetic counterpart in cartilage tissue engineering. Replicating the intricate composition of native cartilage, particularly by incorporating numerous collagen types and essential promoter factors in synthetic bioink, is a challenge. In response, we approached the use of dECM for preserving the native niche and achieving comprehensive biomimetic native cartilage complexity without synthetic alternatives. The findings from bioink CLSM characterisation suggested that the ECM protein composition can be integrated into biomimetic tissue constructs, serving as a potential microenvironment for the proliferation of chondrocytes and BMSCs. A recent investigation of porous injectable micro-carriers (MCs) composed of acellular ECM from cartilage tissue and chitosan demonstrated that MCs with 1% ECM exhibited optimal cell attachment, suggesting that these MCs could be potential candidates for cartilage tissue engineering applications^24^. This finding supports the current work involving the integration of dECM bioink with the biopolymers such as, alginate and with alginate–chitosan composite, representing a strategic 3D approach in cartilage tissue engineering.

The 2D characterisation of chondrocytes and BMSCs-induced chondrocytes revealed isogenous groups, oval-shaped morphology, sulfated GAG accumulation, and the expression of cell-specific and ECM-specific markers. This is indicative of their phenotypic properties as preliminary cell sources for the initiation of 3D biomimetic tissue constructs. Pellet culture with bioink, which was monitored for its increasing pellet size, provided high cellular density, ECM deposition, enhanced BMSCs differentiation and high collagen type II expression, emphasising that pellet culture is the gold standard^25^ for maintaining chondrocytes and BMSCs in a differentiated state^26^. The chondrocytes hypertrophy markers includes collagen type X and matrix metalloproteinases-13 (MMP-13) which inhibit the cell proliferation. Our previous published data on dECM revealed lower levels of MMP-13 and collagen X. Thus the bioink used in the current study amply supports the chondrocytes growth as evidenced in the gene expression and protein expression (Protein Lynx Global Server ratio < 0.5) analysis. Our previous published data also suggested hyaluronan protein 1 and chondroitin sulphate abundance to be more than 50 percent supporting the BMSCs chondrogenesis with bioink^27^.

The hydrophilic nature of alginate beads creates a conducive 3D microenvironment for encapsulating and influencing the behaviour of BMSCs during chondrogenic differentiation. During chondrogenic differentiation, BMSCs first encounter the fibrocartilage-like matrix, a type I collagen fibre that provides differentiation signals to the cell surface. At this stage the cells undergo acclimatisation phase experiencing an initial seven days lag phase. Further interaction with the mixed alginate biopolymer causes BMSCs to undergo condensation, differentiation with rapid exponential growth phase towards a cartilage-like phenotype. During the third week of culture, cells proliferation rate slows down signifying a stable plateau phase of growth and chondrogenesis differentiation^28^. Notably, cell proliferation was observed at the outer edge of the alginate beads, suggesting that cells in this peripheral region experienced greater exposure to the culture medium. These findings have significant implications for improving the outcomes of alginate bead culture. Consequently, the use of smaller beads or a flat alginate substrate may enhance the exposure of more cells to the culture medium^29^.

The alginate 3D beads culture provides a niche for cell–cell and cell–matrix interactions, effectively inducing neocartilage formation with increased collagen type II and aggrecan gene expression when used with bioink additive^30^. Additionally, the alginate biopolymer itself may not directly influence the expression of laminin (a cell adhesion marker), and the 3D environment and bioink additive indirectly support overall tissue-like structural integrity and organization^31^. Recent reports have also highlighted modifications to alginate cultures, including the introduction of layered cultures^32^^.^ Thus, the use of the alginate biopolymer and its introduction as a layer-by-layer technique support our ongoing efforts to optimise composite alginate–chitosan biopolymer under microfluidic systems for cartilage tissue construction.

Chitosan, when used as a bioink in mimicking cartilage, contributes to the structural support of the tissue. It influences the physical and mechanical properties of the hydrogel, impacting its compressibility and stiffness. This supports the growth and proliferation of cells for chondrogenesis. The composite bioink combines the benefits of both alginate and chitosan, aiming to synergise their individual properties. The use of composite hydrogels was supported by the combination of starch-based hydrogels with polyvinyl alcohol (PVA) and borax^33^ or by the addition of nicaraven-loaded chitosan nanoparticles to alginate-based hydrogels^34^, which improved the mechanical properties and enhanced collagen type II gene expression. Similarly, a photocrosslinkable locust bean gum (LBG)-methacrylate (MA) hydrogel that induces chondrogenic differentiation of bone mesenchymal stem cells was reported as a biomimetic ECM for engineering cartilage tissue constructs^35^.

Different combination ratios affect growth factor release kinetics, influencing BMSCs chondrogenic differentiation. Hydrogel preparation with glutaraldehyde concentrations above 1% enhances rigidity and mechanical resistance, while increasing alginate concentration may influence pore size, nutrient diffusion, swelling capacity, degradation rate, and release kinetics^36^. An alginate/cartilage ECM-based injectable hydrogel incorporating silk fibroin nanofibers (SFNs) demonstrated an improved compression modulus with increased alginate concentration (1.6% w/v)^37^. SFN concentration optimisation (1.7% w/v) achieved the desired mechanical stiffness, highlighting the significance of optimising the concentrations of alginate and chitosan at 2% w/v to achieve the optimum compression modulus in alginate–chitosan composite hydrogels. The potential of this composite hydrogel for supporting the development of the organ-on-a-chip (OOAC) model and its mechanical properties are part of our ongoing work.

## Methods

### Ethical statement

Human articular cartilage and bone marrow samples were obtained with prior informed and written consent in accordance with the Declaration of Helsinki and clearance from the Ethics Committee Biomedical Health Research, Gleneagles Global Hospitals (File No: SPF/2021/000126).

### Materials

The cell culture media High glucose Dulbecco's Modified Eagle Medium (H-DMEM,1 g/L D-glucose, 11885-084 500 mL), foetal bovine serum, One Shot™ Format, US origin (FBS, A3160401 50 mL) and collagenase type II powder (1 mg/mL solution, 17101-015) were obtained from Gibco, Life Technologies Corp. HiSep^TM^LSM 1077 (LS001-500 mL) was purchased from HiMedia. L-ascorbic acid (50 µg/mL, A4403), dexamethasone (1 mM, D1756), transforming growth factor beta-2 (TGFβ-2, 50 µg/mL, T2815), basic fibroblast growth factor (bFGF, 10 ng/ml, F3685), sodium alginate powder (2 gm/100 mL, W201502), and chitosan (2%, cat no. 419419-50G) were obtained from Sigma Aldrich. All antibodies were purchased from Novus Biologicals (USA) and used at the recommended dilutions. Antibiotics (gentamycin, 50 IU/ml; amphotericin B, 2.5 µg/ml; penicillin–streptomycin, 100 IU/ml) were purchased from Thermo Fisher Scientific. A 70 µm cell strainer was obtained from Corning® (431751). The forward and reverse primers used were obtained from Eurofins. The remaining chemicals were purchased from Sigma–Aldrich Chemical Co. (USA).

### Methodology

Human bone marrow (2 mL) and articular cartilage explants (1–2 mm) were collected from patients (n = 10) aged 23–70 years as part of a sports injury and knee replacement surgical procedure under aseptic conditions.

### Bioink preparation and sterilisation

Cartilage explants were subjected to mechanical and enzymatic treatment with 0.25% collagenase type II and disrupted further with 2.5% sodium dodecyl sulphate (SDS) for 16 h to obtain cell-free ECM (herein represented as bioink) and achieve efficient decellularisation^27^. The bioink was rinsed with 1 × phosphate-buffered saline (PBS) to remove SDS and further treated with 0.1% Triton X-100 for 5 min to remove nucleic acids. The bioink was further rinsed with 1 × PBS to remove Triton X-100^38^. Before cell seeding, the bioink was sterilized in 1 × PBS solution containing antibiotics (penicillin/streptomycin (500 U/ml) and amphotericin B (0.5 μg/ml)) for 24 h at room temperature to prevent contamination^39^. The sections were then rinsed and stored in 1 × PBS until further use to construct biomimetic tissues.

### Isolation and culture of native chondrocytes

Human articular cartilage explants were collected in H-DMEM and thoroughly rinsed with PBS containing antibiotics to remove undesirable contaminants. Tissue explants were minced using a sterile surgical blade (No. 21), subjected to mechanical and enzymatic digestion with collagenase type II supplemented with 2.5% FBS in H-DMEM supplemented with antibiotics, and incubated for 16 h at 37 °C on an orbital shaker. After incubation, the enzymatic reaction was stopped with 10% FBS, and the cells were strained through a 70 µm cell strainer to separate the chondrocytes from the ECM. The cell suspension was centrifuged at 1800 rpm for 5 min to obtain a cell pellet. The supernatant was discarded, and the cell pellet was rinsed with 1 × PBS by spinning at 1800 rpm for 5 min. The chondrocytes obtained as cell pellet was suspended in H-DMEM consisting of 10% FBS, L-ascorbic acid, dexamethasone, TGFβ-2, and antibiotics^40^ (hereafter referred to as chondrogenic media).

### Isolation and culture of human BMSCs via the density gradient method and differentiation into the chondrogenic lineage

Human bone marrow tissue was collected in an anticoagulant, citrate dextrose (ACD) solution. The tissue was diluted in normal saline at a 1:1 ratio, layered on HiSep solution (1:3 ratio of HiSep to diluted tissue), and subjected to density gradient centrifugation at 1500 rpm for 30 min. The creamy white to buff-coloured layer was collected and centrifuged at 1500 rpm for 5 min. The resulting pellet was rinsed with sterile distilled water to avoid undesirable hematopoietic cell populations and centrifuged at 1500 rpm for 5 min. The cell pellet was suspended in H-DMEM containing 10% FBS, antibiotics and bFGF (BMSCs culture media) and strained through a 70 µm cell strainer to obtain a single-cell suspension^41^. An initial seeding density of 1 × 10^6^ cells/mL was incubated at 37 °C in 5% CO_2_ till 70% confluency was achieved and were subjected to differentiation in chondrogenic media.

### Pellet culture with bioink

Isolated native chondrocytes and BMSCs (6 × 10^6^ cells/mL) along with bioink (~ 100 μL) were seeded in chondrogenic and BMSCs culture media, respectively, and grown as pellet cultures. The cells were pelleted every 3 days at 1800 rpm for 8 min and half replenished with fresh chondrogenic media to retain the growth factors and matrix deposited on the biomimetic tissue constructs. The culture was monitored for an increase in pellet size over three weeks. Simultaneously, a pellet culture without bioink was used as a control.

### Preparation of alginate polymer beads with bioink

Sodium alginate powder (2%) was dissolved in distilled water by stirring on a magnetic stirrer, and the final pH was adjusted to 6.8–7.2. A calcium chloride (100 mM) solution was prepared in 1 × PBS and allowed to dissolve completely to obtain a homogenous solution. The sodium alginate and calcium chloride solutions were filter sterilised with a 0.2 µm syringe filter before alginate bead preparation. Chondrocytes and BMSCs (6 × 10^6^ cells/mL) were suspended in chondrogenic media with sodium alginate solution supplemented with bioink (~ 100 μL), and using a 23-gauge needle, the final suspension was dropped as beads in cold calcium chloride solution. Calcium-alginate beads were incubated at 37 °C for 30 min for polymerisation and encapsulation. The beads were then washed with 1 × PBS and cultured in chondrogenic media^42^. Alginate beads were also prepared and cultured using a similar process without bioink as a control. The cultured alginate beads were paraffin-embedded and sectioned (2 µm thickness) using a microtome for histology and immunocytochemistry (ICC) characterisation.

### In vitro assays

#### Cell viability using a flow cytometer

Chondrocytes and BMSCs-induced chondrocytes (1 × 10^6^ cells/mL) from native and in vitro cultures were stained with 10 µl of 7-AAD cell viability dye, with unstained cells serving as a control. The cells were incubated for 20 min at 4 °C in the dark, and the percentage of live to dead cells among the unstained and stained cells was analysed using a flow cytometer (Becton Dickinson FACS Calibur™).

#### Proliferation assay

To bioink-laden chondrocytes and BMSCs-induced chondrocytes (6 × 10^6^ cells/mL) in H-DMEM without FBS, 5 μM Cell Tracker™ Green CMFDA Dye (Cat. C2925; Invitrogen) was added. The cells were incubated at 37 °C for 30 min and rinsed with 1 × PBS^43^. The cultures were imaged for three weeks at 0, 7, 14, and 21 days using the CLSM software LAS X Office v1.4.5 (Leica Microsystems). The quantitative mean fluorescence values were plotted for biomimetic cultures with and without bioink, and proliferation patterns were analysed.

#### SEM characterisation

The SEM was performed to characterise the cellular attachment and aggregation of the obtained biomimetic tissue constructs without and with bioink. The samples were maintained in a freezer at − 20 °C for 24 h and were lyophilized in a freeze-dryer. The samples were fixed with 4% paraformaldehyde for 30 min at room temperature, followed by rinsing with 1 × PBS three times to remove the fixative. The samples were dehydrated through an ascending concentration of absolute ethanol, starting with 30%, 50%, 70%, 80%, 90%, and 100%, incubated for 30 min time interval and spin at 1200 rpm, 10 min, 4 °C at each step. After the final step of dehydration, another 100% ethanol was added and incubated at room temperature for 30 min. Then, the samples were allowed to dry under high vacuum for 2 h. The samples were mounted on aluminium metal stubs using double-sided adhesive tapes and coated with thin layer (300 angstrom) of ionic gold nanoparticles in sputter coating unit E-1010 (Hitachi Japan) under high vacuum^44^.The gold nanoparticle coating enabled the samples conductive to electrons for capturing images at × 8.0–10.0 k magnification, 15 kilovolts (kV), under high vacuum (10^−7^ torr) using SEM.

#### Histology and ICC characterisation

Histological sections were stained with H&E and alcian blue for morphological identification and accumulation of GAG, respectively. The sections for ICC, were fixed in a 4% paraformaldehyde solution for 5 min, washed 2× with 1 × PBS for 5 min, subjected to antigen retrieval with sodium citrate buffer for 20 min, heated at 95–100 °C, and allowed to cool at room temperature for 20 min. Further blocking and permeabilization were performed using 0.5% Triton X-100 and 5% FBS in 1 × PBS for 20 min at room temperature. The sections were incubated with primary antibodies in a humidifying chamber for 16 h at 4 °C. The primary antibodies used were against CD44 (chondrocyte-specific cellular marker), VCAM1 (differentiated chondrocyte-specific cellular marker), and the ECM-specific markers collagen type I, collagen type II, aggrecan, and laminin to characterise their expression. The primary antibodies were removed by rinsing three times with 1 × PBS for 5 min and incubated with secondary antibodies for 1 h at room temperature on an orbital shaker. Further washes were performed with 1 × PBS, and the sections were counterstained for 5 min with the nuclear stain 4′,6-diamidino-2-phenylindole (DAPI, 1 mg/mL concentration, further diluted to a 1:1000 working stock)^45^. The sections were rinsed with distilled water and mounted with glycerol for imaging using CLSM (Leica TCS SPE).

#### Quantitative PCR (qPCR) gene expression profile

The cultured biomimetic tissue constructs were subjected to RNA isolation using a Qiagen Miniprep Kit (cat. no. 74104), and the quality and purity were checked using a spectrophotometer (NanoDrop2000, Thermo Scientific). The isolated RNA template (2 µg) was subjected to cDNA conversion using 2 × reverse transcriptase (RT) master mix consisting of 10 × RT buffer, 25 × dNTP mixture (100 mM), 10 × RT hexamer random primers, Multiscribe™ RT enzyme, and nuclease-free water in a final reaction volume of 10 µL. The thermocycling conditions were optimized for the initial hold (25 °C, 10 min), RT enzyme activation step (48 °C, 30 min), and final enzyme inactivation step (95 °C, 5 min).

The converted cDNA was subjected to qPCR gene expression profiling using ECM-specific forward and reverse primers for collagen type I, collagen type II, aggrecan, and laminin, with normalization against the housekeeping gene GAPDH (Table 1). qPCR was performed using 5 µg of cDNA added to 2 × SYBR® Select master mix supplemented with the aforementioned forward and reverse primers (10 pmol/µL) in nuclease-free water for a final reaction volume of 20 µL. The PCR cycling and melting curve conditions were set to the initial activation step (95 °C, 5 min), followed by 45 cycles of two-step denaturation (95 °C, 15 s) and annealing/extension (60 °C, 60 s). The final run data were analysed with a threshold value set to 0.05, and gene expression levels and fold changes were calculated using cycle threshold (Ct) values and the 2^−∆∆Ct^ method, respectively^46^.

**Table 1:** Primer sequences for the target ECM genes.

Gene	Primer sequence	Product size (base pair)	NCBI gene reference number
COL1A1	F,5’-GATTCCCTGGACCTAAAGGTGC R,5’-AGCCTCTCCATCTTTGCCAGCA	107	NM_000088.4
COL2A1	F,5’-CCTGGCAAAGATGGTGAGACAG R,5’-CCTGGTTTTCCACCTTCACCTG	149	NM_001844.5
AGGRECAN	F,5’-ACTGGCGAGCACTGTAACAT R,5’-CAATCTCACACAGGTCCCCTT	80	NM_001135.4
LAMININ	F,5’-CGCCATCCAATATCACATGCC R,5’-GTCCAGAGTGATTGTGACCCA	327	NM_005559.4

The table provides the details of the ECM-specific target genes, their forward and reverse primer sequences, which were oriented in the 5’ to 3’ direction, their product sizes in base pairs (bp) and their unique NCBI gene reference numbers for amplification through polymerase chain reaction (PCR).

### Alginate–chitosan composite hydrogel preparation

The alginate–chitosan hydrogel was prepared with various concentrations of sodium alginate and chitosan solutions polymerised with calcium chloride and glutaraldehyde and mixed with and without bioink (Table 2). A 2% chitosan solution was prepared by dissolving chitosan in 1% aqueous glacial acetic acid and heating it in a water bath at 60 °C to obtain a homogenous solution. The sodium alginate solution along with the cell-laden bioink (~ 100 µL) in chondrogenic media was layered over the chitosan solution, followed by layering with a cross-linking solution (1 mL of glyceraldehyde solution mixed in an equal volume of calcium chloride solution) under gentle rotation on an orbital shaker for uniform mixing of the hydrogel. The hydrogel was incubated at 37 °C for polymerisation^47,48^.

**Table 2:** Alginate–Chitosan composite hydrogel.

Sodium alginate (w/v)	Chitosan (w/v)	Glutaraldehyde (v/v)	Calcium chloride	Ratio (alginate: chitosan)	Composite hydrogel
1%	2%	0.5%	100 mM	1:1	
1%	2%	0.5%	100 mM	2:1	
1%	2%	0.5%	100 mM	3:2	
2%	2%	0.5%	100 mM	1:1	
2%	2%	0.5%	100 mM	2:1	
2%	2%	1%	100 mM	2:1	

A comprehensive overview is provided for biopolymers alginate, chitosan and their cross-linking agents glutaraldehyde, and calcium chloride at various concentrations and their composite hydrogel ratios. w/v = weight/volume percentage, v/v = volume/volume percentage, mM = millimolars.

### Statistical analysis

The unpaired two-tailed type t-test statistical analysis was performed for all samples (n = 10) in triplicates using GraphPad Prism software. The mean Ct values were used to calculate standard deviation for each target gene under culture conditions without and with bioink. Samples exhibiting t-test with significant difference in p-value less than 0.05, were considered to be statistically significant, rejecting the null hypothesis.

## Conclusion

This study contributes to the novel approach of advancing in vitro modelling techniques using bioink-laden alginate biopolymer to enhance the phenotype and gene expression of BMSCs-induced chondrocytes toward a more hyaline type cartilage. The integration of bioink additive with composite alginate and chitosan laden chondrogenic lineage studies will be representing a multifaceted approach taken to mimic the native cartilage environment and enhance the prospects of successful cartilage regeneration.

## Data availability 

The submitted manuscript materials, data, and associated protocols will be made available to the readers by the corresponding author (L.K.C) upon request.
